# Satellite- and Epoch Differenced Precise Point Positioning Based on a Regional Augmentation Network

**DOI:** 10.3390/s120607518

**Published:** 2012-06-04

**Authors:** Haojun Li, Junping Chen, Jiexian Wang, Bin Wu

**Affiliations:** 1 Shanghai Astronomical Observatory, Chinese Academy of Sciences, Shanghai 200030, China; E-Mails: yanlhjch@126.com (H.L.); bwu@shao.ac.cn (B.W.); 2 Department of Surveying and Geo-informatics Engineering, Tongji University, Shanghai 200092, China; E-Mail: wangjiexian@tongji.edu.cn

**Keywords:** SDED, differential precise point positioning, regional augmentation, correction information

## Abstract

Precise Point Positioning (PPP) has been demonstrated as a simple and effective approach for user positioning. The key issue in PPP is how to shorten convergence time and improve positioning efficiency. Recent researches mainly focus on the ambiguity resolution by correcting residual phase errors at a single station. The success of this approach (referred to hereafter as NORM-PPP) is subject to how rapidly one can fix wide-lane and narrow-lane ambiguities to achieve the first ambiguity-fixed solution. The convergence time of NORM-PPP is receiver type dependent, and normally takes 15–20 min. Different from the general algorithm and theory by which the float ambiguities are estimated and the integer ambiguities are fixed, we concentrate on a differential PPP approach: the satellite- and epoch differenced (SDED) approach. In general, the SDED approach eliminates receiver clocks and ambiguity parameters and thus avoids the complicated residual phase modeling procedure. As a further development of the SDED approach, we use a regional augmentation network to derive tropospheric delay and remaining un-modeled errors at user sites. By adding these corrections and applying the Robust estimation, the weak mathematic properties due to the ED operation is much improved. Implementing this new approach, we need only two epochs of data to achieve PPP positioning converging to centimeter-positioning accuracy. Using seven days of GPS data at six CORS stations in Shanghai, we demonstrate the success rate, defined as the case when three directions converging to desired positioning accuracy of 10 cm, reaches 100% when the interval between the two epochs is longer than 15 min. Comparing the results of 15 min' interval to that of 10 min', it is observed that the position RMS improves from 2.47, 3.95, 5.78 cm to 2.21, 3.93, 4.90 cm in the North, East and Up directions, respectively. Combining the SDED coordinates at the starting point and the ED relative coordinates thereafter, we demonstrate the performance of RTK PPP with standard deviation of 0.80, 1.34, 0.97 cm in the North, East and Up directions.

## 1. Introduction

It has been more than ten years since precise point positioning (PPP) theory was proposed [1]. PPP has been demonstrated as a valuable technique for single stations positioning over continents, even on a global scale [2,3]. It has been considered an effective tool for precise orbit determination of low Earth orbiting [4], real-time water vapor estimation [5], early warning systems [6] and so on. Usually, the convergence time of traditional PPP requires tens of minutes to achieve the desired centimeter-positioning accuracy [7,8] as the ambiguities in PPP are non-integral and they are conventionally not fixed to integers [9]. The main factors influencing ambiguities fixing in PPP are the non-integral un-calibrated fractional offsets (UFOs) [10,11]. The UFOs are absorbed by the un-differenced ambiguity estimates and their integer properties are thus destroyed [7,12,13]. Integer ambiguity fixing can shorten the convergence time and improve the accuracy of PPP. However, it still takes approximately 15–20 min to achieve the first integer ambiguity solution. In order to further shorten the initialization time, the regional reference network augmented strategy was presented in [14–17], by which the precise atmospheric delay corrections of users are generated by making use of a regional reference network.

The above mentioned strategies focus on the PPP ambiguity resolution. Following the strategy of satellite- and epoch difference (SDED) [8], we differentiate between the observations of adjacent epochs and differences between satellites to remove ambiguities and receiver clocks. As a result, the number of parameters is reduced significantly. The remaining parameters are unknown coordinates and tropospheric delay, where tropospheric delay could be accurately modeled by implementing a interpolation model based on a reference network. On the other hand, the numeric stability of the normal equation is degraded when making epoch differences, because the geometry changes slowly between adjacent epochs. To overcome this problem, we apply the Robust Estimation strategy [18] in our differential PPP. The differential PPP approach thus avoids the ambiguity resolution and residual phase modeling procedure and may improve the robustness of PPP applications. In the following, Section 2 introduces the differential PPP theory and its augmentation method using a regional network; Section 3 introduces the prototype software system used in this study; Section 4 shows the validation results and the discussion; finally, Section 5 summarizes the main points and the conclusions.

## 2. Differential PPP Based on Regional Network

Network augmented PPP (Net-aPPP) [14–17] follows the idea from RTK, where atmospheric delay (troposphere and ionosphere) and other residual errors at user stations could be represented by a model based on the inputs from reference stations. By adding the corrections from the model, the tropospheric and ionospheric and other residual errors are corrected and thus do not appear in the estimation. Consequently, the correlation between parameters is reduced and the PPP solution convergence will be faster. The computation of Net-aPPP is accomplished at user stations and therefore is more flexible and efficient. However, in the above mentioned Net-aPPP approach, the correlation between ambiguities and coordinates still exists. Epoch-differenced (ED) strategy, which removes ambiguities, has been demonstrated to be efficient and accurate in the estimation of satellite clocks [8,19,20]. In GNSS positioning, the ED strategy is generally used to estimate the coordinate difference between adjacent epochs. Li *et al.* [8,21], demonstrated the application of the ED strategy in the absolute PPP positioning. In this Section we first describe the SDED theory, and afterwards the refinement of SDED approach by applying augmentation corrections is discussed.

### 2.1. SDED Based Ambiguity-Free Equation

Assuming that the residual errors of satellite orbit and clock could be neglected, the ionosphere-free (*L_3_*) combinations can be written as follows:

(1) $$L^{j}=\rho^{j}+\delta +b^{j}+T^{j}+{ɛ}^{j}$$

The superscript of “***j***” indicates tracked satellite. ***L****^j^* is the ionosphere-free phase observation of ***j***; *ρ^j^* is geometric range between receiver and satellite; ***δ*** is the receiver clock **error**; ***b****^j^* is the ambiguity of ***L****_3_* phase observation; *T^j^* is the tropospheric delay; *ε^j^* is the phase noise of ***L****_3_* observation.

For one receiver tracking two satellites (***j,i***) simultaneously, the single-differenced measurements between satellite can be used to eliminate the receiver clocks. By taking differences of ***L****_3_* observation of satellite ***i*** and ***j***, we obtain:

(2) $$L^{j,i}=L^{i}-L^{j}=\rho^{j,i}+b^{j,i}+T^{j,i}+{ɛ}^{j,i}$$

Equation (2) is the defined as the satellite-differenced (SD) equation. Assuming there are no cycle slips between two adjacent epochs, the ambiguity term *b^j,i^* in Equation (2) can be further eliminated by differencing the SD observations at the adjacent epoch ***n*** and ***n−1*** (***n =** 2…****n****_1_*, ***n****_1_* is total number of defined epochs):

(3) $$\Delta L^{j,i}(n)=L^{j,i}(n)-L^{j,i}(n-1)=\Delta \rho^{j,i}(n)+\Delta T^{j,i}(n)+\Delta {ɛ}^{j,i}(n)$$

where “Δ” indicates the ED operator. Equation (3) can be re-written as:

(4) $$\Delta \rho^{j,i}(n)=\Delta L^{j,i}(n)-\Delta T^{j,i}(n)-\Delta {ɛ}^{j,i}(n)$$

Equation (4) is defined as the SDED based ambiguity-free equation.

### 2.2. Network Based Corrections of Tropospheric Delay and Un-Modeled Error

Although the main parts of the tropospheric delay are canceled by forming differences between adjacent epochs, there are still residual components in Equation (4). Due to the correlation between parameters, it is difficult to obtain a quick and reliable estimation of both coordinates and tropospheric parameters. A better solution is to model tropospheric delay rather than to estimate it. Following the RTK method, the modeling of atmospheric delay has been studied by a large number of researchers [22–24]. The general idea is the interpolation based on a reference network. The atmospheric delays at reference stations could be accurately estimated while they could be modeled at user stations. In practice, the wet part of tropospheric delay (Zwd) of user stations is interpolated, while the dry part (Zhd) is corrected using a standard Saastamoinen model. The interpolation model [23] is:

(5) $$\text{Zwd}=\sum_{m=1}^{k}(Zwd_{m}\cdot P_{m})/\sum_{m=1}^{k}P_{m}$$

(6) $$P_{m}=\frac{1}{l_{m}}$$

where *k* is the number of reference stations; *Zwd_m_* is Zwd of reference station *m; P_m_* is weight; *l_m_* is the distance between the reference and user stations.

Mathematically, the SDED method is sensitive to the epoch-wise un-modeled errors (UMEs). These errors include satellite related errors (orbits and clocks) and appear to be similar at a regional scale. At reference stations, we could calculate the remaining un-modeled errors. With estimated tropospheric delay, fixed coordinates of the reference station, clocks and orbits, the SDED UMEs of reference stations can be retrieved from the SDED observations. Similar to Equation (5), the SDED UMEs at user station can be computed based on the inverse distance weighted model:

(7) $${\text{UME}}^{j,i}(n)=\sum_{m=1}^{k}({\text{UME}}^{j,i}{\left(n\right)}_{m}\cdot P_{m})/\sum_{m=1}^{k}P_{m}$$

### 2.3. Coordinate Estimation

Substituting the interpolated tropospheric delay corrections and computed UMEs into Equation (4), and linearizing Equation (4), Equation (4) can be re-written in a matrix form:

(8) $$\left(a^{j,i}(n-1)-a^{j,i}(n))\cdot X=\Delta l_{0}^{j,i}(n\right)$$

where *a^j,i^(n − 1)* and *a^j,i^(n)* are SD design matrices. The unknowns are the coordinates of the user station. From Equation (8), we know that two epochs of observations are sufficient to get coordinate estimations when there are more than three observation equations (*i.e.*, four satellites are observed simultaneously).

The coordinate estimation using Equation (8) could be used as the starting point for kinematic positioning thereafter, *i.e.*, after observing in static mode for a short period, a user station could run kinematically afterwards. The strategy for user kinematic PPP positioning is as following: SDED starting points + epoch-wise coordinates differences. In this strategy, the SDED coordinates from Equation (8) are used as known coordinates and epoch-wise coordinates differences (relative coordinates) are derived following the ED described in Bock *et al.* [4]. The final absolution coordinates are epoch-wisely accumulated using the initial and relative coordinates. The precision of relative coordinates is normally at mm level, therefore the precision of kinematic coordinates is similar to that of the starting points.

## 3. Realization of Network Based Differential PPP

**Figure 1.**
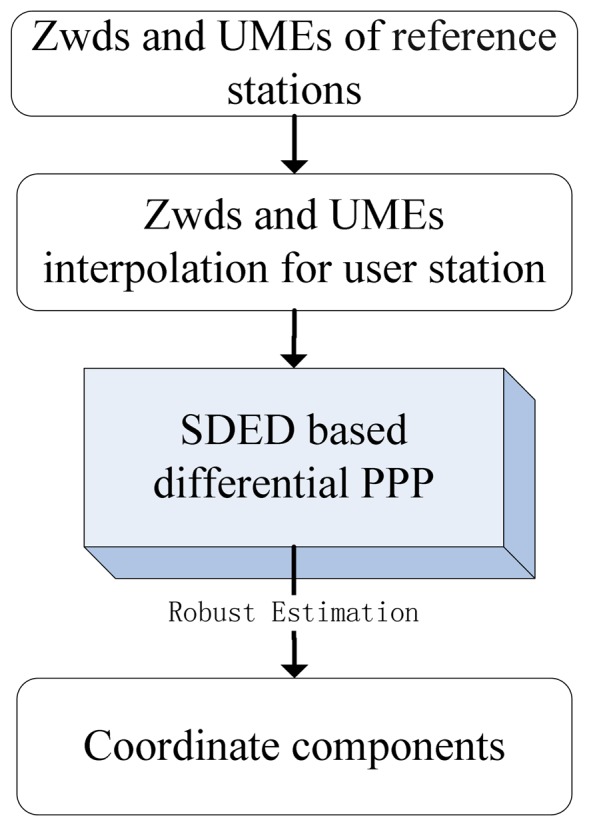
Flow chart of the SDED based differential PPP software.

According to Equation (8), the precise coordinate estimations of user station could be obtained by applying the corrections of tropospheric delay and un-modeled error. A prototype software for the SDED based differential PPP was developed. The flow chart of the software is shown in Figure 1.

The preprocessing of phase data is based on the Melbourne-Wuebbena and Geometry-free combination to detect cycle slips and outliers. For real-time applications, the orbit and clock are from the predicted ultra-rapid orbits and real-time estimated clocks. The correction models including the phase wind-up, Earth tides, relativistic effects, antenna phase center offset and variation, *etc.* are implemented according to IERS convention 2003 [25] or IGS recommendation. At reference stations, the Saastamoinen model [26] is used to get the *a priori* correction and the wet part is estimated with an interval of 1 h. The Zwds at user stations are interpolated using the Zwds which are estimated by setting up a Pice-Wise-Constant (PWC) parameter at an interval of 1 h at reference stations.

The robust estimation [18] is used in parameter estimation procedure, where for each observation the weight function is as follows:

(9) $$p_{i}=\{\begin{array}{cc} 1 & \left|V_{i}\right|\leq k_{0}\\ \frac{k_{0}}{\left|V_{i}\right|}{\left(\frac{k_{1}-\left|V_{i}\right|}{k_{1}-k_{0}}\right)}^{2} & k_{0}<\left|V_{i}\right|\leq k_{1}\\ 0 & \left|V_{i}\right|>k_{1} \end{array}$$

where *p_i_* is the weight; *V_i_* = *v_i_*_/_σ*_0_* is a factor showing quantity of each residual *v_i_*, σ*_0_* is the calculated variance factor; *k_0_* and *k_1_* are defined constants which can be chosen by experiment or by the actual observation distribution. Following Yang [18], we chose *k_0_* as 1.5 and *k_1_* as 3.0.

## 4. Experimental Validations

**Figure 2.**
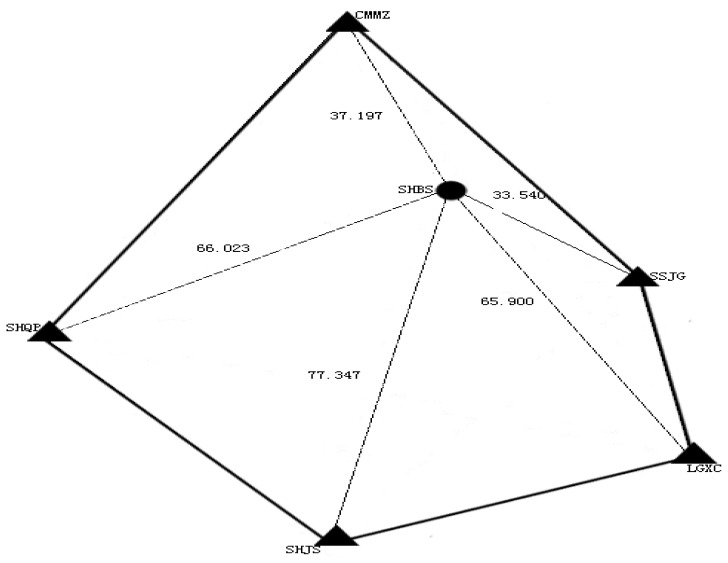
Stations selected from Shanghai CORS and their distribution. Solid triangle shows the reference stations, Circle is user station.

In order to test the proposed method, one week (DOY 011 to DOY 017, 2009) data from six stations (SHBS, CMMZ, SHQP, SHJS, LGXC and SSJG) of Shanghai Continuous Operation Reference Station (CORS) network are used. Each station has 12-h observations for each day. The station SHBS is taken as user station and the other five stations are used as reference stations to derive the correction information. Data sampling is 30 s and elevation cut-off angle is set to 9 degrees. The reference and user stations are shown in Figure 2. The averaged inter-station distance is about 56 km.

### 4.1. Interpolated Zwds

**Figure 3.**
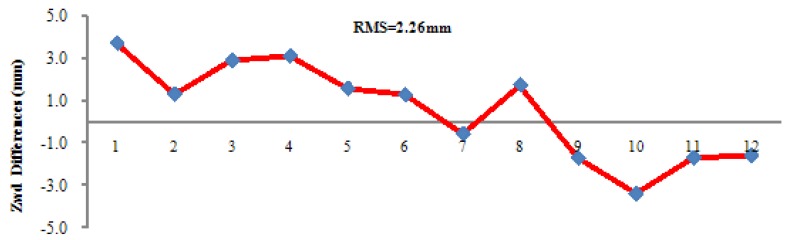
Differences between estimated and interpolated Zwds of SHBS.

Taking the Zwds estimated at the five reference stations, the Zwds of the station SHBS are interpolated with the method described in Section 2.2. Comparison between estimated and interpolated Zwds is illustrated in Figure 3. In Figure 3, the Zwds of all stations (including SHBS) are first estimated by setting up a Pice-Wise-Constant (PWC) parameter at an interval of 1 h. Afterwards, Zwds of SHBS are interpolated based on the estimated Zwds of the other stations and are compared to the previous estimation. The RMS is 2.26 mm.

### 4.2. Retrieved Un-Modeled Errors

**Figure 4.**
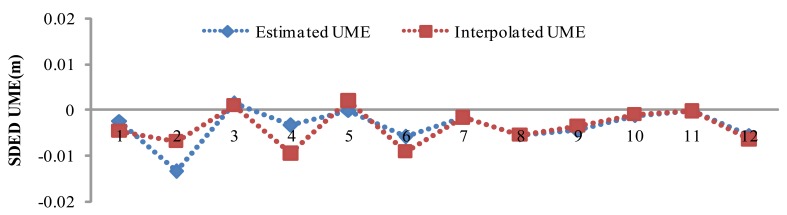
SDED un-modeled errors of SHBS using satellite pair of PRN20 and PRN28, where Estimated UME is calculated following Equation (4) and Interpolated UME is derived based on the 5 reference stations following Equation (7).

The SDED UMEs of the all the stations can be retrieved using Equation (4). Using the retrieved SDED UMEs at the five reference stations, SDED UMEs at SHBS are interpolated according to Equation (7). Figure 4 illustrates for satellite pair PRN20 and PRN28 the retrieved SDED UMEs in 2 h with an interval of 10 min. Comparing the interpolated and estimated UMEs, we found very good agreement with RMS of 3 mm, which validates the idea of UMEs interpolation of user station.

**Figure 5.**
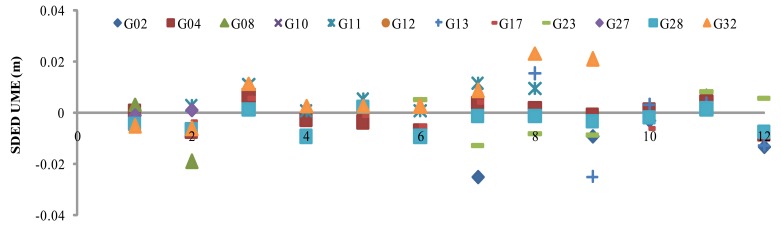
Satellite- and epoch differenced un-modeled errors.

Figure 5 illustrates the SDED UMEs for all satellites with PRN20 being the reference satellite. It is observed that SDED UMEs are sometimes more than 2 cm, e.g., PRN02, PRN04, PRN10, PRN13, PRN17 and PRN32.

### 4.3. Success Rate

SDED-based differential PPP was performed for SHBS for the whole week. As two epochs of data are sufficient to derive station coordinates, we split the 12-h observations into 72 10-min and 48 15-min sessions for each day. Each session thus contains only two epochs of data with the interval at 10-min or 15-min. Differential PPP was carried out to verify the robustness and efficiency of the proposed SDED approach. The estimators of Least Square (LS) and Robust Estimation (RE) were tested by different strategies. The difference between strategy 1 and 6 is in the tropospheric delay handling: it is corrected using models in strategy 1, while it is being estimated in strategy 6. Table 1 presents for each tested strategy the success rate of differential PPP under the sampling of 10 min and 15 min. The success rate is defined as the case that the difference between the estimated and the known coordinate components are less than 10 cm. For comparison, the traditional PPP (NORM-PPP) method was tested using the observations (sampling at 30 s) within the same time window and the results were listed in Table 1 as well.

The success rate shown in Table 1 of different strategies indicates that the residual tropospheric delays affect the positioning results, although they can be partly eliminated by the epoch and satellite difference. The study strategy 1 is actually the normal SDED PPP [21], its success rate is only 36% due to the correlation between tropospheric parameters and coordinates. Applying the tropospheric delay corrections, the success rate of LS improves from 36% to 52%. Another improvement of 6% (in case of LS) and 32% (in case of RE) of success rate is achieved when we further apply the UME corrections. This improvement validates the contribution of UME estimation. Comparing the results of Robust Estimation to that of Least Square estimation, we see that success rate has notable improvement as bad SDED observations are down weighted in the robust estimation. Comparing the success rate of 10 min and 15 min, there is a general 10% improvement in success rate when the interval between the two epochs is increased to 15 min. By correcting tropospheric delay and UMEs, all sessions implementing differential PPP successfully meet the threshold and reach the desired accuracy.

### 4.4. SDED Positioning Accuracy

Position RMSs are calculated based on results from sessions with 10-min and 15-min intervals. Table 2 shows for each tested strategy the RMS (in cm) of coordinates with respect to the known coordinates in the North, East and Up directions at the interval of 10 min and 15 min, respectively.

Similar to the success rate discussed in previous section, the position results show also the improvement due to the corrections of tropospheric delays and UMEs. From the results, we see RE performs better than LS in all cases with the best result improved from 2.47, 3.95, 5.78 cm to 2.21, 3.93, 4.90 cm in the North, East and Up directions, respectively. Similarly, the cases where UME corrections are applied behave better than the others. All the cases of differential PPP have a better precision than NORM-PPP with the best strategy (strategy 6) has around 50% improvement in all three coordinate components.

### 4.5. Kinematic Positioning

**Figure 6.**
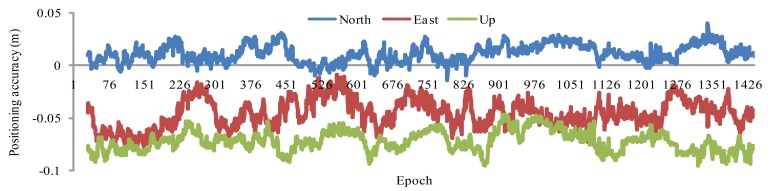
SDED & ED combined kinematic PPP positioning.

To demonstrate the RTK applications of our new SDED approach, SHBS is used as a kinematic station. We use the first RTK strategy described in Section 2.3 to derive kinematic PPP coordinates. The first two epochs of data sampling at 15 min is used to derive coordinates at the starting point, data sampling is then set to 30 s in RTK PPP thereafter. Figure 6 shows the RTK coordinates differences for DOY 011 with respect to the known coordinates. We notice that there are offsets of few centimeters for each component, which are introduced by the SDED initial coordinates. Removing the offsets, the coordinate standard deviation (STD) is of 0.80, 1.34, 0.97 cm in the North, East and Up directions.

## 5. Discussion and Conclusions

This study introduces a differential PPP approach based on a regional reference augmentation network, where ambiguity and receiver clock are removed by the SDED model. The corrections of tropospheric delay and UME at user station are interpolated from the estimated Zwds and retrieved UMEs of reference stations. In addition, the Robust Estimation is implemented to overcome the defect of the weakened geometry caused by the SDED model.

The approach presented in the paper has been validated using one-week data from a regional network in Shanghai. From the experiment, we see the interpolated Zwds and UMEs have an accuracy of 2.26 mm and 3 mm, respectively. Using the interpolated corrections, differential PPP is performed with estimators of Least Square and Robust Estimation. The position results and success rate indicate that the RE performs better than the LS. The PPP position accuracy of RE is at the cm level for an interval longer than 10 min. The success rate of RE with an interval longer than 15 min reaches 100% and position accuracy reaches 2.21, 3.93, 4.90 cm in the North, East and Up directions. Comparing the results from our new approach to that of NORM-PPP, we see remarkable improvements in both convergence and precision.

The differential PPP approach improves PPP positioning with shorter convergence-time and more reliable results. In real-time applications, the user station observes firstly in static mode for around 15 min and runs kinematically afterwards. Results show the kinematic PPP coordinates precision (STD) is of 0.80, 1.34, 0.97 cm in the North, East and Up directions, with offsets of a few centimeters in each component.

## Figures and Tables

**Table 1.** Strategies, Success rate (in %) within 10-min and 15-min time window.

**Estimator**	**Strategy**	**Correction**	**Success rate**	

			**10-min**	**15-min**

LS	1	No	36	27
	2	Zwd	52	63
	3	UME and Zwd	58	77

RE	4	Zwd	70	81
	5	UME and Zwd	85	100

NORM-PPP	6		46	60

**Table 2.** RMS (in cm) of different strategies static PPP coordinates with respect to the known coordinates in the North, East and Up directions.

**Estimator**	**Corrections**	**10 min**			**15 min**		

		**North**	**East**	**Up**	**North**	**East**	**Up**

LS	Zwd	7.68	10.19	12.47	4.13	5.96	6.20
	UME and Zwd	7.49	9.11	11.35	2.47	3.95	5.78

RE	Zwd	6.11	8.43	10.02	2.44	3.82	5.62
	UME and Zwd	5.35	6.50	8.12	2.21	3.93	4.90

NORM-PPP		7.93	10.23	12.56	4.51	7.86	8.91
